# Ultra‐Rapid and Specific Gelation of Collagen Molecules for Transparent and Tough Gels by Transition Metal Complexation

**DOI:** 10.1002/advs.202302637

**Published:** 2023-09-11

**Authors:** Tomoyuki Suezawa, Naoko Sasaki, Yuichi Yukawa, Nazgul Assan, Yuta Uetake, Kunishige Onuma, Rino Kamada, Daisuke Tomioka, Hidehiro Sakurai, Ryohei Katayama, Masahiro Inoue, Michiya Matsusaki

**Affiliations:** ^1^ Division of Applied Chemistry, Graduate School of Engineering Osaka University 2‐1 Yamadaoka Suita Osaka 565–0871 Japan; ^2^ Joint Research Laboratory (TOPPAN) for Advanced Cell Regulatory Chemistry, Graduate School of Engineering Osaka University 2‐1 Yamadaoka Suita Osaka 565–0871 Japan; ^3^ Innovative Catalysis Science Division, Institute for Open and Transdisciplinary Research Initiatives (ICS‐OTRI) Osaka University 2‐1 Yamadaoka Suita Osaka 565–0871 Japan; ^4^ Department of Clinical Bio‐resource Research and Development Kyoto University Graduate School of Medicine Kyoto 606–8304 Japan; ^5^ Division of Experimental Chemotherapy, Cancer Chemotherapy Center Japanese Foundation for Cancer Research Tokyo 135‐8550 Japan

**Keywords:** 3D‐cell culture, collagen, healing property, rapid gelation, transparency

## Abstract

Collagen is the most abundant protein in the human body and one of the main components of stromal tissues in tumors which have a high elastic modulus of over 50 kPa. Although collagen has been widely used as a cell culture scaffold for cancer cells, there have been limitations when attempting to fabricate a tough collagen gel with cells like a cancer stroma. Here, rapid gelation of a collagen solution within a few minutes by transition metal complexation is demonstrated. Type I collagen solution at neutral pH shows rapid gelation with a transparency of 81% and a high modulus of 1,781 kPa by mixing with K_2_PtCl_4_ solution within 3 min. Other transition metal ions also show the same rapid gelation, but not basic metal ions. Interestingly, although type I to IV collagen molecules show rapid gelation, other extracellular matrices  do not exhibit this phenomenon. Live imaging of colon cancer organoids in 3D culture indicates a collective migration property with modulating high elastic modulus, suggesting activation for metastasis progress. This technology will be useful as a new class of 3D culture for cells and organoids due to its facility for deep‐live observation and mechanical stiffness adjustment.

## Graphical abstract

Specific gelation of collagen molecules for construction of transparent gels with controlling elastic modulus from tens to over mega pascals by transition metal complexation within a couple of minute is discovered for the first time. This technology has a great potential for 3D culture of cancer cells and organoids due to its deep‐live observation and easy adjusting of elastic modulus functions.

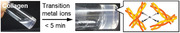

## 1 Introduction

Extracellular matrices (ECMs) contain/possess/are made of key components for regulating the cell microenvironment, controlling cell functions such as adhesion, migration, growth, differentiation, cell‐cell interaction, protein secretion, and metabolism.^[^ ^1^, ^2^ ^]^ Collagen (Col) is an important fibrillar ECM as it is a tension‐resisting element which maintains tight and stable tissue structures.^[^ ^3^ ^]^ Col is a main ECM component of connective (stromal) tissues including tumors.^[^ ^4^ ^]^ Recently, increase in ECM stiffness caused by increasing the secreted amount of Col has been found to be one of the strongest risk factors for cancer progression.^[^ ^5^, ^6^ ^]^ For example, although normal breast has a stiffness of only 0.8 kPa, nodal metastasis rates of 396 breast cancer patients ranged from 7% for tumors with a mean stiffness of < 50 kPa to 41% for tumors with a mean stiffness of > 150 kPa.^[^ ^7^ ^]^ Moreover, increased the elastic modulus of colorectal cancer tissues of 106 patients showed a strong correlation with T stage, denoting the size and extent of the main tumor, and the median values of the elastic moduli at T1 and T4 stages were 2.81 (max. 3.96) and 13.8 (max. 68.0) kPa, respectively.^[^ ^8^ ^]^ The reason for these stiffness increases depending on cancer progression has been clarified as increasing Col secretion.^[^ ^5^, ^6^ ^]^ Accordingly, cytocompatible control of the elastic modulus of Col hydrogels for the 3D culture of cancer cells has been keenly sought after.

To prepare Col hydrogels, covalent bond formation has been generally formed by using a chemical crosslinker such as glutaraldehyde (GA)^[^ ^9^ ^]^ or 1‐ethyl‐1‐3‐(3‐dimethylaminopropyl) carbodiimide hydrochloride (EDC)‐*N*‐hydroxysuccinimide (NHS),^[^ ^10^ ^]^ an enzyme such as transglutaminase^[^ ^11^ ^]^ or peroxidase,^[^ ^12^ ^]^ and UV irradiation.^[^ ^13^ ^]^ However, the elastic modulus of the obtained gels is not high and the crosslinkers show cytotoxicity. Genipin has been reported as a cytocompatible crosslinker,^[^ ^14^ ^]^ but the obtained gel is stained blue by the reacted genipin derivatives. There have been a few reports on physical crosslinkers, but the elastic moduli of the obtained gels were low.^[^ ^15^ ^]^ To avoid the cytotoxicity issue of crosslinkers, a physical crosslinking method by neutralization and heating of a collagen acidic solution has been used as a standard method of 3D‐cell culture. Recently, Lama and co‐workers reported the fabrication of tough collagen gels by a method of concentrating collagen microparticles.^[^ ^16^ ^]^ Salameh et al. discovered transparent tough collagen materials by finding a narrow range of collagen concentration that inhibited light scattering.^[^ ^17^ ^]^ Although these reports are promising, their application in 3D‐cell culture is challenging. Tough Col hydrogels for 3D‐cell culture with over tens kPa modulus have generally not been constructed because of the low dissolving property of Col molecules into an aqueous solution at neutral pH condition for cell culture. Accordingly, Col hydrogels below 1.0 wt% concentration are commonly used for 3D culture of cancer cells so their elastic modulus is lower than 1.0 kPa.^[^ ^18^ ^]^ We recently reported a unique approach, “sedimentary culture”, using a collagen microfiber (CMF) to fabricate large‐scale engineered tissues.^[^ ^19^, ^20^, ^21^ ^]^ The millimeter‐sized tissues with high ECM density were easily obtained by centrifugation of cells and CMFs, but the maximum elastic modulus of the obtained tissues was ≈10 kPa, much lower than that of cancer at the late stage.

Another limitation of Col gel culture is observation because, although it is a standard method of 3D‐cell culture, the gels cannot be observed clearly at great depth even by confocal laser scanning microscopy (CLSM) due to their opaque color. Although transparent collagen gels have been reported in the field of cornea,^[^ ^22^, ^23^ ^]^ 3D‐cell culture in the transparent gels has yet to be achieved. Live observation at great depth is still a major challenge in Col gel culture.

In this study, we demonstrate for the first time the rapid gelation of Col solutions by transition metal complexation. Transition metal ions form a complex between a nitrogen or oxygen atom of inter/intra Col molecules, whereas basic metal ions do not. Although interaction between *cis*‐diamminedichloroplatinum (II) (cisplatin), which is a well‐known anticancer agent, and a Col model peptide has been reported,^[^ ^24^ ^]^ there are no reports on crosslinking of natural Col fibers to rapidly form a gel by complexation with transition metal ions. The elastic modulus of the obtained transparent Col gels is widely controllable by varying the metal ion concentration up to 1.8 MPa. Furthermore, we found that live imaging of a patient‐derived colon cancer organoid showed activated morphology of the collective migration at 162 kPa, which is a higher modulus range than that of a colon cancer at the T4 stage. This rapid‐gelation technology for modulus‐controllable and transparent Col gels will provide a new class of 3D‐cell cultures for drug assessment and basic cancer biology.

## 2 Results and Discussion

**Figure 1:**
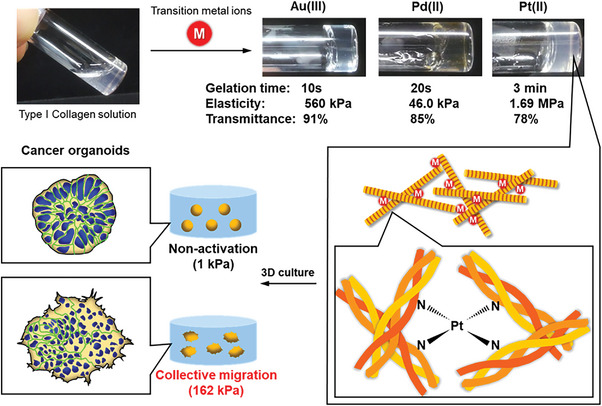
Structural illustration of Col I‐TM gels. CNF solution is quickly crosslinked by the coordinate bonding of transition metal ions to nitrogen or oxygen atoms of collagen molecules, resulting in a transparent gel with high elasticity. The modulus‐controllable properties of this gel are useful for mechanobiology assays with 3D cultures of cancer organoids.

**Figure 2:**
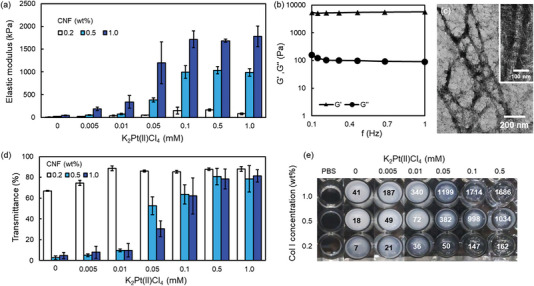
Characterization of Col‐Pt gels. a) Elastic moduli of Col‐Pt gels constructed under 0.005–1.0 mM K_2_Pt(II)Cl_4_ and 0.2–1.0 wt% CNF conditions at r.t. (n = 3). b) Viscoelasticity of Col‐Pt gels constructed at 0.5 mM K_2_Pt(II)Cl_4_ and 1.0 wt% CNF at 37 °C. c) TEM images of Col‐Pt gels constructed at 0.1 mM K_2_Pt(II)Cl_4_ and 0.2 wt% CNF at 37 °C. The gels were stained with uranyl acetate. d) Transmittance at 500 nm and e) photograph of the obtained Col‐Pt gels in 96 microwell at 37 °C (n = 3). The values in each well are elastic moduli (kPa).

Type I collagen nanofibers (CNFs)^[^ ^25^ ^]^ which undergo a reversible sol‐gel transition from 4 °C to 37 °C under neutral pH conditions were used in this study to avoid the influence of pH on gelation because conventional type I collagen needs to be dissolved in an acidic solution at 4 °C and then both neutralization and heating are necessary for its gelation. When K_2_Pt(II)Cl_4_ solution was added to the CNF solution under a stirring condition, the solution quickly solidified (within 3 min) and the stirring bar was completely stopped (**Figure** **1**, Figure S1a and Video S1, Supporting Information), suggesting intermolecular crosslinking of CNFs by K_2_Pt(II)Cl_4_ The obtained gels were completely transparent and stably maintained even after flipping the bottle. The elastic modulus of the obtained gels increased with increasing K_2_Pt(II)Cl_4_ and CNF concentrations and reached a plateau at 0.1 mM, independent of CNF concentration (**Figure** **2**a). Surprisingly, the elastic moduli evaluated by compression tests exceeded 1.0 MPa even in a wet gel condition and the maximum value was 1.78 MPa in the 1.0 wt% CNF and 1.0 mM K_2_Pt(II)Cl_4_ condition. Even the lowest 0.2 wt% CNF condition yielded 162 kPa at 0.5 mM K_2_Pt(II)Cl_4_, a much higher value than that of the conventional 1.0 wt% Col hydrogels with a 1.0 kPa modulus prepared by neutralization and heating.^[^ ^18^ ^]^

Interestingly, almost all water‐soluble transition metal ions such as group 10 (Ni(II), Pd(II), and Pt(II)/(IV)), group 11 (Cu(II) and Au(III)), and group 12 (Zn(II)) showed rapid gelation with a high elastic modulus (Video S2, Supporting Information). Conversely, group 8 (Fe(III)), group 9 (Co(II) and Ir(IV)), and part of group 11 (Ag(I) and Au(0)) did not show this phenomenon which was the same as group 2 (alkaline earth metal: Mg(II), Ca(II), and Ba(II)) (Table S1, Supporting Information). From these data, at least multivalent transition metal ions were expected to have the rapid gelation property of Col I molecules. The resulting Col‐transition metal (TM) gels were named “Col‐TM gels”.

The rheological property of the Col‐Pt gels is shown in Figure 2b. Storage modulus (G’) was stably maintained at ≈5,500 Pa under frequency variation, suggesting high surface stiffness. Loss moduli (G’’) were 100–150 Pa, ≈50–fold lower than the G’ value, and Tan δ (G’’/G’) were 0.016–0.025, indicating a solid‐like, typical chemically bonded property.^[^ ^26^ ^]^ The reason why the G' values are much lower than the elastic modulus of the compression test in Figure 2a is that the rheological analysis was performed immediately after mixing CNF and K2PtCl4 to prevent complete gelation. Once the Col‐Pt gels completely formed, rheological analysis could not be performed due to slipping or stopping of a cone at the gel surfaces. TEM observation of Col‐Pt gels indicated D‐band periodicity in the small‐sized collagen fibrils (<100 nm in diameter), but few cross‐striated larger‐sized collagen fibers were observed (Figure 2c), suggesting that the main networks of the gels are composed of the small‐sized collagen fibrils. This could be one of the reasons for the lack of scattering in the Col‐TM gels.

To gain an understanding of the transparency of the Col‐Pt gels, their transmittance was measured at different K_2_Pt(II)Cl_4_ and CNF concentrations (Figure 2d). The gels prepared with 0.2 wt% CNF solution all showed transparency greater than 67%. The gel transparency increased with increasing Pt ion concentration, reaching a plateau of 90% at a Pt ion concentration above 0.01 mM. The gels prepared with higher CNF concentrations also showed the same trend, reaching a plateau of 80% at a Pt ion concentration above 0.5 mM even though their elastic moduli were higher than 1 MPa (Figure 2e). CNF gradually formed turbid gels under the condition without K_2_PtCl_4_ at 37 °C during 1 day of incubation, and the micrometer to millimeter‐sized aggregates of collagen molecules in the gels by phase‐separation scattered light strongly. However, K_2_PtCl_4_ immediately formed CNF gels within a couple of minutes, and such a rapid crosslinking at molecular level can prevent aggregation formation at 37 °C. This is the reason why the transparency of the gels increased with increasing K_2_PtCl_4_ concentration. The homogeneous structures of Col‐Pt gels without phase separation were also confirmed by the observation of optical coherence tomography (Video S4, Supporting Information). The above findings suggest the formation of a homogeneous network inside the gels.^[^ ^27^ ^]^

**Figure 3:**
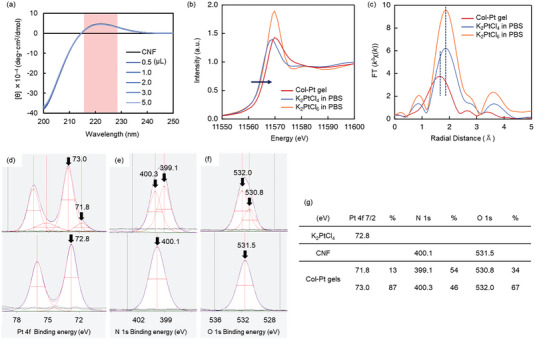
Structural analysis of Col I‐Pt gels. a) CD spectra of a CNF solution within different concentrations of Pt(II) at room temperature. b) XANES and c) EXAFS of K_2_PtCl_4_ and K_2_PtCl_6_ in PBS and Col‐Pt gels. XPS spectra of d) Pt 4f_7/2_ of Col‐Pt gel (top) and K_2_Pt(II)Cl_4_ powder (bottom), e) N 1s of Col‐Pt gels (top) and CNF gels (bottom) and f) O 1s of Col‐Pt gels (top) and CNF gels (bottom), respectively. The Col‐Pt gels were prepared by 50 mM Pt(II) ions and 0.2 wt% CNF. CNF gels were prepared using 0.2 wt% CNF solution by incubation at 37 °C overnight. g) Binding energy peak shift of (d) Pt 4f_7/2_, (e) N 1s and (f) O 1s of Pt(II) ions, CNF, and Col‐Pt gels prepared by 50 mM Pt(II) ions and 0.2 wt% CNF.

In general, Col gels composed of triple‐helix molecules have an opaque color due to phase‐separation, while gelatin gels composed of the denatured collagen molecules with a random structure have transparent properties. To confirm the triple‐helix structures of the Col‐TM gels, circular dichroism (CD) spectra of the gels using HAu(III)Cl_4_ were analyzed under different metal ion concentrations (Figure S1, Supporting Information). The CNF exhibited a typical positive peak at 225 nm corresponding to a triple‐helix structure, and the peak intensity showed no change with increasing TM ion concentration, suggesting that the triple‐helix structure was fully maintained by this gelation. Since the Col‐Pt gels prepared by K_2_Pt(II)Cl_4_ also indicated the same triple‐helix CD spectra (**Figure** **3**a), the crosslinking by TM ions did not induce the denaturation of the collagen molecules.

To understand the role of Col gelation, other commercially available types of collagen molecules such as type II, III, and IV from different animal species were mixed with K_2_Pt(II)Cl_4_ in the same way as for Col I (Table S2, Supporting Information). All collagen molecules in phosphate buffered saline (PBS) at pH 7.4 quickly gelled with the similar transparency (78–85%) as the CNF solution, suggesting the importance of the triple‐helix structure for Pt crosslinking. Furthermore, other types of ECMs, collagen model peptides, polysaccharides, synthetic polymers, and DNA were selected to evaluate their potential for gelation by Pt ions (Table S3, Supporting Information). Although gelatin is a component of the single collagen α chain in the triple helix, it did not show gelation, suggesting the importance of triple‐helix molecules (Video S3, Supporting Information). A collagen model peptide ((POG)_10_) had a collagen triple‐helix structure with a positive peak at 225 nm in the CD spectra but did not show gelation probably due to its much lower molecular weight. DNA has a double‐helix structure but yielded the same results. Laminin and fibronectin are well known cell adhesive ECMs with the same high molecular weight as collagen, however gelation was not observed. Natural polysaccharides, heparin and alginate, and the synthetic polymer polyacrylate were also used because of their abundance of oxygen atoms that can be expected to form coordinate bonds with Pt ions. However, none of the molecules exhibited gelation in the same manner as collagen molecules. These results strongly suggest that this method reveals huge and rigid molecule selectivity of the collagen triple helix. The difference between DNA and the collagen triple helix is interesting because both have a rigid helical structure and DNA is well known for its ability to make coordinate bonds with cisplatin Pt(II).^[^ ^28^ ^]^ The triple helix of collagen is a more linear and packed molecule than DNA because it self‐assembles to form collagen fibrils. Thus, dense packing of the huge molecules is considered to be important.

Because gelatin molecules dissolve well in water and partly form a triple‐helix structure at the edge of the molecules in a high concentration condition, higher concentrations of both gelatin and K_2_Pt(II)Cl_4_ were used as shown in Table S4 (Supporting Information). Gelatin with a high concentration of >10 wt% showed gelation when mixed with 10 mM K_2_PtCl_4_ solution, probably due to partially formed triple helical structure packing. To understand molecular entanglement, the viscoelasticity of both Col I and gelatin were measured at different concentrations (Table S5, Supporting Information). Interestingly, Col I showed ≈20‐fold higher viscoelasticity than gelatin(comparison between 0.2 wt% CNF and 5.0 wt% gelatin: both are the lowest concentration of Pt gelation), probably due to intermolecular hydrogen bond formation of huge linear collagen nanofibrils.^[^ ^3^ ^]^ These results clearly suggest the importance of tight packing huge collagen fibrils for TM ion crosslinking.

To confirm the local geometry around platinum atom in Col‐Pt gels, Pt L_3_‐edge X‐ray absorption spectroscopy (XAS) was performed. Col‐Pt gel prepared from K_2_Pt(II)Cl_4_ was incubated for 1 week at 4 °C to form the tough gel, and XAS experiments were conducted with fluorescent method. Focusing on the X‐ray absorption near edge structure (XANES) region, the absorption edge of the Col‐Pt gel appeared at 11 565 eV, which is ≈1 eV higher than that of K_2_Pt(II)Cl_4_ in PBS (Figure 3b). Since the first peak of Pt L_3_‐edge XAS corresponds the electric dipolar transition from filled 2p_3/2_ to unfilled 5d orbitals, the observed chemical shift denotes the increase of 5d energy level of platinum. Therefore, this result suggested the ligand exchange from the chloride atoms to electron‐donating elements, such as oxygen or nitrogen included in collagen structure, occurred during the gelation process. In addition, the peak intensity of Col‐Pt gel was almost same as that of K_2_Pt(II)Cl_4_, and lower than that of K_2_Pt(IV)Cl_6_, indicating that the platinum atom in Col‐Pt gel possesses a square‐planer geometry which is typical for Pt(II) complexes. The extended X‐ray absorption fine structure (EXAFS) of Col‐Pt gel, K_2_Pt(II)Cl_4_, and K_2_Pt(IV)Cl_6_ were shown in Figure 3c. In the case of K_2_Pt(II)Cl_4_ and K_2_Pt(IV)Cl_6_, a peak was observed at 1.9 Å which corresponds the Pt–Cl scattering. Meanwhile, the peak was shifted to 1.6 Å after gel formation. This result indicates that the nearest elements of platinum were replaced by light elements, such as oxygen and nitrogen, showing a good agreement with the result of XANES data. To sum up, the platinum in the Col‐Pt gels has a square planar structure and is found to bind to nitrogen and oxygen atoms present in collagen. Hence, it is thought that the platinum plays a role in connecting the collagen fibers having triple‐helix structure to form a cross‐linked network as shown in Figure 1, that is considered to be an origin of the toughness of the thus‐fabricated Col‐Pt hydrogels. Noted that the XANES spectra of Pt(II) in Col‐Pt gels after 1 h and 1 week of incubation were almost the same, indicating that 1 h incubation was sufficient for ligand exchange (Figure S2, Supporting Information).

For further confirmation of the connection between Pt(II) and N or O atoms in collagen molecules, the X‐ray photoelectron spectroscopy (XPS) analysis was conducted (Figures 3d–g). The 4f orbitals of Pt are split by spin‐orbit coupling, and the binding energy of 4f_7/2_ core level was used to evaluate the electric state.^[^ ^29^ ^]^ Although the peak for K_2_Pt(II)Cl_4_ powder was a single peak at 72.8 eV which was the same as in a previous report,^[^ ^29^ ^]^ two peaks at 71.8 and 73.0 eV were estimated by curve fitting, suggesting the exchange of ligands from Cl atoms to the other atoms (Figure 3d). The peak shift to lower binding energy denotes a decrease in electrical binding from the nucleus, suggesting the exchange from Cl to N or S (higher to lower electronegativity).^[^ ^30^ ^]^ On the other hand, the peak shift to higher energy denotes the exchange from Cl to O (lower to higher electronegativity). The peak splitting was also observed on N 1s XPS in CNF gels (Figure 3e). The peak shift from 400.1 eV to 399.1 eV indicates the existence of polarized Pt^δ+^─N^δ–^ bonds, suggesting the complexation with Pt atoms. Similar peak shift was also found on O 1s XPS in CNF gels (Figure 3f). The results of XPS spectra summarized in Figure 3g clearly indicated the bond formation between Pt(II) and N and O atoms in collagen molecules.

To measure the amount of Pt(II) ions used for crosslinking to form the Col‐TM gels, inductively coupled plasma (ICP) spectroscopy was conducted (Figure S3, Supporting Information). The amount of Pt(II) ions inside the gels increased with increasing CNF concentration, while excess Pt(II) ions in washing buffers decreased, suggesting an increase in the crosslinking bonds inside the gels by Pt(II)‐collagen coordinate bonds (Figure S3a, Supporting Information). In the case of increasing Pt(II) ion concentrations, the Pt(II) ions inside the obtained gels gradually increased and then reached a plateau at concentrations exceeding 0.5 mM Pt(II) ions (Figure S3b, Supporting Information). The theoretical crosslinking bond number in the Col‐TM gels prepared by 0.5 wt% CNF and 0.5 mM K_2_Pt(II)Cl_4_ was calculated as 2.4 × 10^−7^ by the 12 µg of Pt(II) ions because Pt(II) ions form tetragonal coordinate bonds. Amino acids that could possibly be crosslinked in collagen molecules by Pt(II) ions are expected to be hydroxyproline, serine, tyrosine, threonine, histidine, lysine, hydroxylysine, asparagine, aspartic acid, glutamine, glutamic acid, methionine, and cysteine based on the results of XPS spectra indicating Pt–N and Pt–O formation (Figures 3d–g) and previous reports on Pt–S formation.^[^ ^30^ ^]^ Since the ratio of these amino acid residues in collagen molecules was estimated at 321:1000,^[^ ^31^, ^32^ ^]^ the theoretical maximum crosslinking bond in the gels is estimated to be 5.1 × 10^–6^, ≈21‐fold higher than the above estimated number, probably due to the steric hindrance of huge linear collagen fibrils and segregation of the possible amino acids to the fiber interior.

**Figure 4:**
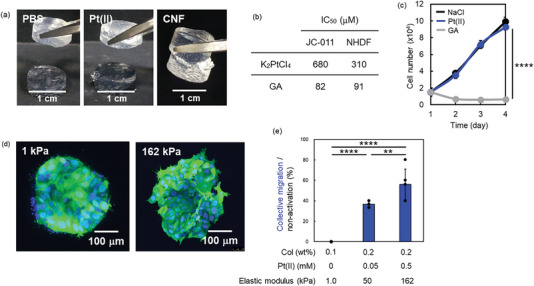
a) Healing property on the cleavage surfaces of Col‐Pt gels constructed with 1.0 wt% CNF and 1.0 mM Pt(II) by adding a further1.0 wt% CNF. b) IC_50_ values of K_2_Pt(II)Cl_4_ and GA against a patient‐derived colon cancer cell line (JC‐011) and NHDFs in DMEM containing 10% FBS with varied concentrations after 3 days of culture at 37 °C (n = 3). The values were calculated from Figure S4 (Supporting Information). c) Cell proliferation profiles of NHDFs in DMEM containing 10% FBS with 0.1 mM NaCl, GA, or K_2_Pt(II)Cl_4_ for 4 days of culture (n = 3). d) CLSM live imaging of Hoechst staining for 3D cultured EGFP‐labeled organoids in stiff (162 kPa) Col‐Pt gels constructed by 0.2 wt% CNF with 0.5 mM K_2_PtCl_4_ solutions. The control soft gels (1 kPa) without Pt (II) ions were constructed by a commercial 0.1 wt% type I collagen solution by neutralization and heating at 37 °C. e) Mean percentage of both collective migration and non‐activation of the organoids after 3D culture inside the gels constructed by varied Col and Pt(II) concentrations (n = 5–8). Statistical comparisons between groups were analyzed by one‐way ANOVA. A *p* value, * <0.05, ** <0.01, ***<0.001, and ****<0.0001 were statistically significant.

For the application of the Col‐TM gels, the self‐healing property at the cleavage surface was expected because of the noncovalent nature of the coordinate bond as previously reported.^[^ ^33^ ^]^ Unfortunately, however, the cleavage surfaces of the Col‐TM gels did not attach well even after overnight incubation. We therefore added additional solution of PBS, 1.0 mM K_2_Pt(II)Cl_4_ and 1.0 wt% CNF at the cleavage interfaces and the gels were then incubated at r.t. for 1 h (**Figure** **4**a). In the case of PBS and K_2_Pt(II)Cl_4_, the adhesive phenomenon was not observed at all. However, in the solution with CNF, adhesion of the cleavage surfaces was clearly observed within 10 min, which might have been due to excess Pt(II) ions, as shown in Figure S3 (Supporting Information). Additional Col molecules in between the cleavage surfaces were crosslinked by the excess Pt(II) ions, because the crosslinkable amino acid residues in the Col molecules seemed to be already wholly crosslinked during the Col‐TM gel formation.

Finally, the cytocompatibility of the Col‐TM gels was evaluated to understand their potential application in cancer cell culture. Patient‐derived colon cancer cell line (JC‐011) and normal human dermal fibroblasts (NHDFs) were cultured in dulbecco's modified eagle medium (DMEM) containing 10% fetal bovine serum (FBS) and K_2_Pt(II)Cl_4_ or glutal aldehyde (GA) at varied concentration for 3 days, and then half maximal inhibitory concentration (IC_50_) of Pt(II) ions and GA was estimated (Figure 4b; Figure S4, Supporting Information). GA was used as a control of typical chemical crosslinker. IC_50_ values of Pt(II) ions to JC‐011 and NHDFs were 680 and 310 µM respectively and these were 8.2 and 3.4‐fold lower cytotoxicities than those of GA, suggesting the potential of the Col‐TM gels for 3D cell cultures at high elastic modulus. To understand the effect of Pt(II) ions to cell growth, cell proliferation profiles of NHDFs were evaluated for 4 days in DMEM containing 10% FBS with 0.1 mM NaCl, K_2_Pt(II)Cl_4_ or GA, respectively (Figure 4c). Pt(II) ions revealed exactly the same growth profile as NaCl, but GA caused a decrease in cell number, suggesting strong cytotoxicity. These data clearly demonstrate that Pt(II) ions have high potential for 3D‐cancer cell cultures with controllable elastic modulus.

To understand the stiffness effect of the collagen‐gel matrix to the phenotype of cancer cells, patient‐derived colorectal cancer (CRC) organoids were sandwich‐cultured by the Col‐TM gels at a stiff (162 kPa) elastic modulus for 7 days and observed by CLSM in live conditions (Figure 4d). We previously reported a cancer tissue‐originated spheroid (CTOS) method, in which cell‐cell contact of cancer cells were maintained throughout the preparation process.^[^ ^34^ ^]^ The organoids prepared by CTOS method is useful for drug screening and personalized therapy because it retains the properties of cancer cells in the patient's original tumor.^[^ ^35^, ^36^ ^]^ When CRC organoids were cultured in control soft collagen gels at 1 kPa, no morphological changes were observed and the cancer cells remained uniformly aligned. However, the 162 kPa Col‐TM gels clearly indicated a marked spike and cell migration toward the outer boundary, suggesting morphology of collective migration that is the process by which a group of cells moves together, without completely disrupting their cell‐cell contacts. To understand the stiffness effect in detail, the concentration of CNF and K_2_Pt(II)Cl_4_ solutions was varied for 3D culture of the organoids and the ratio of collective migration was estimated from the organoid morphology (Figure 4e; Figures S5 and S6, Supporting Information). The concentration of K_2_Pt(II)Cl_4_ solutions was adjusted to <1.0 mM to avoid cytotoxicity. When concentration of K_2_Pt(II)Cl_4_ solutions was increased, the ratio of the collective migration clearly increased regardless of Col concentration. This suggests that collective migration may be induced by the stiffening of the ECM scaffolds in 3D culture. The collective migration is important during morphogenesis, and in pathological processes such as wound healing and cancer cell invasion.^[^ ^37^ ^]^ While the molecular mechanisms of collective migration are becoming clear, analysis of the underlying mechanisms of collective migration in cancer is still at an early stage. The importance of mechanical cues in the collective migration has recently been studied, and in vivo experiments have revealed that tissue stiffening initiates the epithelial‐to‐mesenchymal transition that triggers collective migration.^[^ ^38^ ^]^ Although biomechanical methods such as 2D culture, micro‐patterning, 3D‐tube migration, and computational simulation have been reported extensively,^[^ ^39^ ^]^ there are no reports of collective migration initiated by stiffening the ECM scaffolds in 3D culture. The 3D culture in the Col‐TM gels with controllable environmental ECM stiffness has the potential to accelerate in vitro analysis of the underlying mechanism of collective migration in cancer.

## 3 Conclusion

We reported the rapid gelation of Col solutions by transition metal complexation with nitrogen or oxygen atoms of inter/intra Col molecules. It has been reported that chrome treatment improves the heat resistance of tanned leather, and the complexation of collagen fibrils and chromium was proposed as the mechanism, but the details remained unclear.^[^ ^40^ ^]^ This study reveals the historical mechanism of complexation of transition metal ions with collagen fibers and shows a new application of collagen transparent gelation in the biomedical field. This stiffness‐controllable, rapid, and transparent gelation technique may be useful for a new class of biomaterials for tissue engineering and basic biology.

## 4 Experimental Section

### Preparation of CNF

Pepsin‐treated Type I collagen (Col I) sponge were kindly donated by NH Foods Ltd (Osaka, Japan). The 50 mg of type I collagen sponge and 5 ml of PBS (D5652‐10L, Sigma–Aldrich, St. Louis, USA) were added into a 15 ml centrifuge tube (Corning, 430 791, NY, USA). A φ8 mm blade was set in a homogenizer (AS ONE, VH‐10 1‐8471‐31, Osaka, Japan) and homogenized collagen sponge at a speed of 6 (30,000 rpm) for 6 min. After homogenization, the collagen suspension was incubated at 4 °C for 1 day to dissolve collagen microfibers. After incubation, the solution was centrifuged (5922, KUBOTA, Tokyo, Japan) at 25 °C and 10 000 rpm for 3 min to remove bubbles and CNF solution. Other concentrations were prepared in the same manner by changing the amount of collagen sponge or adjusting the concentration after resuspension.

### Gelation of CNF Solution by Transition Metal Ions

Five hundred µl of 0.5 wt% CNF solution and 20 µl of 12.5 mM potassium tetrachloroplatinum (II) acid (K_2_PtCl_4_: 206075‐1G, Sigma‐Aldrich, St. Louis, USA) in PBS to the sample tube (0201‐01 Maruemu, Osaka, Japan). Then, they were mixed using a pipetter (SA05825, Gilson, Middleton, USA) for high viscous solution, 0.5 mM Pt(II)−0.5 wt% CNF gel was prepared at 4 °C for 1 day. For the following metal species, gels were prepared in the same manner and the concentration of each metal was adjusted by the amount of metal solution. Magnesium chloride (MgCl_2_: 136–03995, WAKO, Osaka, Japan), Calcium chloride (CaCl_2_: 039–00475, WAKO, Osaka, Japan), Barium (II) chloride (BaCl_2_: 026–00185, WAKO, Osaka, Japan), Iron (III) chloride (FeCl_3_: 010–40684, KISHIDA CHEMICAL, Osaka, Japan), Cobalt (II) chloride (CoCl_2_: 035–10982, WAKO, Osaka, Japan), Iridium (IV) chloride (IrCl_4_: 092–02921, WAKO, Osaka, Japan), Nickel (II) chloride (NiCl_2_: 654507‐5G, Sigma–Aldrich, St. Louis, USA), Palladium (II) chloride (PdCl_2_: 7647‐10‐1, TCI, Tokyo, Japan), Cis‐diamine dichloroplatinum (II) (Pt(NH_2_)Cl_2_: 039–20093, WAKO, Osaka, Japan), Potassium hexachloroplatinum (IV) (K_2_PtCl_6_: 206067‐1G, Sigma–Aldrich, St. Louis, USA), Copper (II) chloride (CuCl_2_: 032–04142, WAKO, Osaka, Japan), Silver (I) nitrate (AgNO_3_: 000–70555, KISHIDA CHEMICAL, Osaka, Japan), Gold nanoparticles (Au NP: 752 568, Sigma–Aldrich, St. Louis, USA), Gold (III) acid tetrahydrate (HAuCl_4_: 077–00931, WAKO, Osaka, Japan), and Zinc (II) chloride (ZnCl_2_: 261–00272, WAKO, Osaka, Japan).

Gelation was visually confirmed from naked‐eye observation after tilting the bottles. The classification of gelation condition was as follows: the circle means complete gelation, the triangle means partly gelation, and the x means non‐gelation. The CNF gels by each metal species were prepared in 24 well inserts (Corning, clear‐3470, NY, USA) and then the elastic moduli of the obtained gels were measured after 1 day of incubation at 37 °C.

Colorectal cancer surgical specimens were collected from patients who underwent surgery of primary and metastasized colorectal tumor. The patients submitted written informed consent for genetic and biological analyses, which were performed in accordance with the protocols approved by the institutional review board (IRB) of Japanese Foundation for Cancer Research (#2013‐1093).

XAS measurements were performed at the BL‐9A of KEK under the approval of the Photon Factory Program Advisory Committee (proposal no. 2020G006).

## Conflict of Interest

The authors declare no conflict of interest.

## Supporting information

Supporting Information

Supplementary Video S1

Supplementary Video S2

Supplementary Video S3

Supplementary Video S4

## Data Availability

Research data are not shared.
