# Supplementary material for: Ultra‐Rapid and Specific Gelation of Collagen Molecules for Transparent and Tough Gels by Transition Metal Complexation
Source: Adv Sci (Weinh). 2023 Sep 11;10(30):2302637. doi: 10.1002/advs.202302637 (PMC10602541; doi:10.1002/advs.202302637)
Supplement: Supplementary file 1 — Supporting Information [file ADVS-10-2302637-s003.pdf]

ADVANCED SCIENCE
Open Access

# Supporting Information

for *Adv. Sci.*, DOI 10.1002/advs.202302637

Ultra-Rapid and Specific Gelation of Collagen Molecules for Transparent and Tough Gels by Transition Metal Complexation

*Tomoyuki Suezawa, Naoko Sasaki, Yuichi Yukawa, Nazgul Assan, Yuta Uetake, Kunishige Onuma, Rino Kamada, Daisuke Tomioka, Hidehiro Sakurai, Ryohei Katayama, Masahiro Inoue and Michiya Matsusaki**

Supporting Information

# Ultra-Rapid and Specific Gelation of Collagen Molecules for Transparent and Tough Gels by Transition Metal Complexation

*Tomoyuki Suezawa, Naoko Sasaki, Nazgul Assan, Yuta Uetake, Kunishige Onuma, Rino Kamada, Daisuke Tomioka, Hidehiro Sakurai, Ryohei Katayama, Masahiro Inoue, and Michiya Matsusaki**

**Contents of supporting information:**

**S1. Materials and methods**
**S2. Effect of TM species on gelation**
**S3. S-S curve of Col-Pt gels and CD spectra of Col-Au gels and denatured collagens**
**S4. Effect of collagen species and type on gelation**
**S5. Effect of polymer type on gelation**
**S6. Effect of gelatin concentration on gelation**
**S7. Viscoelasticity of CNF and gelatin solutions**
**S8. Pt $L_3$-edge X-ray absorption spectroscopy of Col-TM gels**
**S9. ICP data of Col-Pt gels**
**S10. Cell viability assay**
**S11. 3D-culture of organoids in Col-Pt gels**
**S12. Gelation videos of Col-TM gels**
**S13. OCT observation**
**S14. References**

## S1. Materials and Methods

### Gelation of polymers and proteins solution by transition metal ions

To evaluate the gelation properties of the following polymers and proteins, the same gelation procedure using 0.5 wt% polymer in phosphate buffer solution (PBS, pH=7.4) and 0.5 mM $K_2PtCl_4$ was performed.

Pig skin type III collagen (Col III: KP-5005, Nitta Gelatin, Osaka, Japan), Pepsin-treated and untreated porcine skin type I atero- and tropo-collagens (Col I and Tropo Col I: donated by NH Foods Ltd), Human Col I (donated by NH Foods Ltd), Human placental type IV collagen (Col IV: C7521, Sigma–Aldrich, St. Louis, USA), Chicken cartilage type II collagen (Col II: donated by NH Foods Ltd), Bovine dermis Col I (ASC-1-100-100PW, Nippi, Tokyo, Japan), collagen mimic peptide: $(POG)_{10}$ (4033, Peptide Institute INC., Osaka, Japan), Laminin (354259, Corning, NY, USA), Fibronectin (F2006-5MG, Sigma-Aldrich, St. Louis, USA), Heparin (081-00136, WAKO, Osaka, Japan), Alginic acid (180947-100G, Sigma-Aldrich, St. Louis, USA), Polyacrylic acid (169-18591, WAKO, Osaka, Japan), DNA from salmon sperm (043-31381, WAKO, Osaka, Japan), and Gelatin (077-03155, WAKO, Osaka, Japan) were used in this study.

Gelation of 5.0–20 wt% gelatin solution was also evaluated by $K_2PtCl_4$ at 1–50 mM. In addition, the viscosities of 0.2–1.0 wt% CNF and 5.0–20 wt% gelatin solutions were measured using a Rheometer (HAKKE RheoStress 6000, Thermo Scientific).

### Circular Dichroism (CD) Spectroscopy

The CD spectra were acquired on a spectrometer (J-725, JASCO, Tokyo, Japan) and using a quartz cuvette with 10 mm path length. The 0.5–5.0 µl of 12.5 mM $K_2PtCl_4$ and $HAuCl_4$ solutions were added in 3 mL of 0.005 wt% CNF in PBS for CD spectrum measurement. Mean residue ellipticity (MRE, deg·cm$^2$·dmol$^{-1}$) values were calculated using the following equation, where $\theta$ is the ellipticity (deg), $l$ is the path length (m), $C$ is the collagen and gelatin concentration (M), $N$ is the number of residues.

$$\mathrm{MRE} = \frac{\theta}{C \times l \times N}$$

### X-ray Absorption Spectroscopy (XAS)

Pt $L_3$-edge X-ray absorption spectroscopy (XAS) experiments were performed at the BL-9A of KEK using Si(111) double-crystal monochromatized synchrotron radiation under the ring-conditions of 2.5 GeV and 450 mA. All XAS experiments were carried out using fluorescent method at room temperature. The ionization chamber was used to measure the intensity of the incident X-ray ($I_0$). The X-ray fluorescence was monitored with a 7-element silicon drift detector (SDD). XAS analyses were conducted using Demeter package, a comprehensive system for processing and analyzing X-ray absorption spectroscopy data.[S1–S3] Background removal and normalization of raw data were performed by cubic spline method using Athena software. $E_0$ was

defined as photon energy at the absorption edge where μT = 0.5 in the normalized μ(*E*) spectrum. The $E_0$ value of the Pt foil was set to 11564 eV for photon energy calibration. The extracted $k^3$-weighted EXAFS oscillation was Fourier transformed in the *k*-range of 3.0–8.0 $Å^{-1}$.

To a vial (0201-01, Maruemu, Osaka, Japan) containing 1.0 wt% CNF, $(POG)_{10}$, or gelatin in PBS (pH = 7.4, 1.92 mL) was added a PBS containing Pt ion ($K_2PtCl_4$ or $K_2PtCl_6$) (5 mmol/L, 80 μL). After incubating for 60 min or 1 week at 4 °C, the thus-prepared hydrogel or solution was transfer to a plastic bag (polypropylene), and subjected to XAS measurement.

**X-ray photoelectron spectroscopy (XPS)**

The Col-Pt dried gel films prepared by $K_2PtCl_4$ were used for XPS measurements. 0.2 M Pt(II)/PBS solution was prepared and then mixed with 0.2 wt% CNF solution in a 1.5 ml tube (3810X, Eppendolf, Hamburg, Germany) to prepare 50 mM Pt(II)-0.2 wt% CNF solution. Before gelation, 50 µL of mixed solution was rapidly dropped onto a cover glass (C218181, MATSUNAMI, Osaka, Japan) and then dried at 37 °C for overnight. For standard of XPS measurements, 50 µl of Au-NPs (752568, Sigma–Aldrich, St. Louis, USA) in PBS was dropped onto all dried samples and dried at 37 °C for 3 hours. The films were fixed onto the XPS stage with a conductive double-sided carbon tape and then measured on a X-ray photoelectron spectrometer (JPS-9010, JEOL, Tokyo, Japan) using X-ray source of Mg.

**Inductively coupled plasma (ICP)**

The Col-Pt gels were prepared in 0.1–1.0 mM Pt(II)-0.5 wt% CNF in PBS and 0.5 mM Pt(II)-0.2-1.0 wt% CNF in PBS by $K_2PtCl_4$ in 24 well inserts. Each gel was immersed in 10 ml of PBS for 1 day at room temperature and then washed with fresh 10 ml of PBS. The total 20 ml of supernatant were collected and lyophilized to obtain as a powder. The obtained powders were completely dissolved in 5 ml of 0.1 M HCl. The obtained Col-Pt gels were minced and then completely dissolved in 2 ml of 5 M HCl in 24 well plate (3820-024, IWAKI, Shizuoka, Japan). One ml of the obtained solution was transferred to a 15 ml tube and diluted with 4 ml of 0.1 M HCl. The supernatants and dissolved solutions were transferred to glass tubes (TEST15-105NP, IWAKI, Shizuoka, Japan) and then an ICP atomic emission spectrometer (ICPS-7510, SHIMATZU, Kyoto, Japan) was used to determine the amount of Pt(II) ions inside the gels or supernatants using calibration curve of 0–10 ppm Pt(II)/0.1 M HCl solution.

**Compression test**

The elastic moduli of the obtained Col-TM gels were measured using a compression tester (EZ-Test, SHIMADZU, Kyoto, Japan). CNF solutions were incubated at 37 °C for 1 day in 24 well insert after mixing with transition metal solution, and then immediately subjected to compression testing at room temperature. The elastic modulus *E* (kPa) was calculated using the following equation, where *S* is the slope of the stress-strain curve (mN/mm), *H* is the height of the gel (mm)

and $C$ is the contact area of the testing jig with the gel ($mm^2$)

$$E = \frac{S \times H}{C}$$

The slope of the graph was selected to follow the rising edge of the stress-strain curve at strains between 5-10 %, the height of the gel was measured using a digital caliper (Digimatic Caliper CD-15CP, Mitsutoyo, Kanagawa, Japan), and the contact area of the jig was calculated by integrating the strain values in the stress-strain curve.

**Rheological analysis**

500 µl of 1.0 wt% Col-Pt gel just after mixing with 0.5 mM $K_2PtCl_4$ was added onto a rheometer and the moduli were measured on a plate geometry with a 20 mm cone at a gap of 0.5 mm using frequency sweep mode in the range of 0.1–1 Hz at 37 °C of stage temperature with 10 Pa stress.

**TEM observation**

Col-TM gels were constructed by mixing 0.2 wt% CNF and 0.1 mM $K_2Pt(II)Cl_4$ at r.t. for 10 min. The constructed gels were placed on Cu grids and then dried in vacuum at r.t. for overnight. After drying, the gels were washed with Milli-Q for twice and then the dried samples were stained with 2% uranium acetate. The gels were observed using a transmission electron microscope (H-7000, HITACHI, Tokyo, Japan).

**Transmittance measurement**

0.005-1.0 mM Pt(II)-0.2-1.0 wt% Col-Pt gels were prepared by $K_2Pt(II)Cl_4$ in 96 well plate (3860-096, IWAKI, Shizuoka, Japan) by incubation at 37 °C for 1 day. To evaluate the transmittance, absorbance at 500 nm was measured using a plate reader and transmittance was calculated using following equation. $A$ means the absorbance at 500 nm.

$$\text{Transmittance}(\%\text{T}) = 100 \times 10^{-A}$$

**Optical coherence tomography observation**

The Col-Pt gels constructed by 1.0 wt% CNF solution mixed with 0.5 mM $K_2Pt(II)Cl_4$ at r.t. for 1 day were observed by optical coherence tomography (OCT) at the region of 4 $mm^2$ area and 1.35 mm depth. Scanning pitch is 5 µm and exposure time is 300 µsec.

**Patient selection and establishment of patient-derived colon cancer cell line (JC-011)**

Colorectal cancer surgical specimens were collected from patients who underwent surgery of primary and metastasized colorectal tumor. The patients submitted written informed consent for genetic and biological analyses, which were performed in accordance with the protocols approved

by the institutional review board (IRB) of Japanese Foundation for Cancer Research (#2013-1093). Several pieces of the surgically resected tumors were immediately transferred into the ice-cold culture medium with antibiotic-antimycotic (Gibco). Tumor tissues were cut into small fragments, and enzymatically digested with collagenase/dispase (Roche) and DNase I in StemPro hESC culture medium (Invitrogen) for 60 minutes. After washing with antibiotic–antimycotic and 0.2% BSA-containing PBS, the cell pellets were cultured in the StemPro hESC medium supplemented with 10 μmol/L of Y-27632 to establish the patient-derived JC-011 cell line. Before subsequent experiments, the cells were subcultured in the 1:1 mixed medium of RPMI1640 and Ham's F-12 supplemented with 10 % fetal bovine serum (FBS), 1 % antibiotic–antimycotic and 10mM Hepes buffer until the coexisting stromal cells were scarcely detected under the microscope.

**Cytotoxicity assay**

Normal human skin fibroblasts (NHDFs) and patient-derived colon cancer cell line (JC-011) were seeded at 1.0 x $10^4$ cell numbers on collagen-coated 96 well plates and cultured for 1 day at 37 °C and 5 % $CO_2$ condition using the 200 μl of Dulbecco modified Eagle's medium (DMEM: 08458-16, nacalai tesque, Kyoto, Japan) or the 1:1 mixed medium of RPMI1640 (189-02025, WAKO, Osaka, Japan) and Ham's F-12 (087-08335, WAKO, Osaka, Japan), respectively. Both media were supplemented with 10 % fetal bovine serum (FBS) (10270-106, Thermo Fisher Scientific, MA, USA) and 1 % antibiotics. The 0.2 M $K_2PtCl_4$, NaCl, or glutaraldehyde (020-34385, KISHIDA CHEMICAL, Osaka, Japan) in PBS was diluted to 0.01–5.0 mM by each culture medium. After the medium removing and rinsing the cells with PBS, 200 μl of each fresh medium containing the substances was added, and subsequently incubated for 1 day. After the incubation, the culture media were removed and then the cells were rinsed with 100 μl of fresh media and subsequently the cell viability was estimated by a WST-8 reagent (07553-15, nacalai tesque, Kyoto, Japan). To evaluate effect of the substances on cell proliferation, NHDFs were seeded at 1.0 x $10^4$ cell numbers in 48 well plates (3830-048, IWAKI, Shizuoka, Japan) and incubated in 500 μl of DMEM for 1 day at 37 °C and 5 % $CO_2$ condition. After the medium removing and cell rinsing with PBS, 500 μl of fresh DMEM containing substances was added for 1–3 days of culture. The living cell number was estimated by a WST-8 reagent at each time point.

**Healing property of Col-Pt gels**

To evaluate self-healing property of Col-Pt gels, 1 ml of 0.5 mM Pt(II)-1.0 wt% CNF gel was prepared in a circular mold using $K_2PtCl_4$. After cutting the gels in half, 20 μl of PBS, 1.0 mM $K_2PtCl_4$ in PBS, or 1.0 wt% CNF solution was added to the cut gel surfaces and then the gels were attached together for 1 day of incubation at room temperature. The photos were taken after lifting the gels.

**Culture of cancer tissue-originated spheroid**

The clinical features of the patients from whom human CRC organoids were derived have been described in a previous report[34]. The organoids were prepared according to the CTOS method[32]. In brief, resected xenograft tumors were mechanically dissociated, then partially digested with 0.26 U/ml of Liberase DH (Roche, Mannheim, Germany). The organoids were collected using 100- and 40-μm cell strainers (BD Falcon, Franklin Lakes, NJ, USA). The organoids were cultured in StemPro hESC (Invitrogen, Carlsbad, CA, USA).

**Plasmid construction and gene transfer to the organoids**

Organoids were transfected with the expression vector pPiggyBac (PB)-Ubc.eGFP-neo and pCMV-hyPBase. Electroporation was performed in 2-mm gap cuvettes at 150 V for 5 msec, using Type II NEPA21 electroporator (Nepa Gene, Chiba,Japan). After transfection, organoids were selected with G-418 (Roche Applied Science) and maintained in the medium containing G-418.

**3D-culture of organoids in Col-Pt gels**

50 μl of 0.5 mM Pt(II)-0.2 wt% CNF solution was added in 24 microwells and then incubated at 37 $^{o}$C for 30 min to prepare the bottom gels. Five of the EGFP-labeled organoids were added to 50 μl of 0.5 mM Pt(II)-0.2 wt% CNF solution and then dropped on the surfaces of the bottom gels surfaces. After incubation at 37 $^{o}$C for 30 min to fabricate assembled Col-Pt gels containing the organoids, 2 ml of StemPro hESC (Invitrogen, Carlsbad, CA, USA) was added and then incubated it for 7 days. The gels were incubated with 1 μg/mL propidiumiodide (PI) (Molecular Probes) and 2 μg/mL Hoechst33342 (Molecular Probes) at 37 °C for 15 min to visualize the morphology of the organoids and the dead cells. After washing the gels with Hank's balanced salt solution (HBSS, Merck, Darmstadt, Germany), the morphologies of over 10 organoids were observed by Leica DMi8 microscope (Leica Microsystems, Wetzlar, Germany) and then the organoid shapes were categorized for the following two types.

1) There are clear boundaries, and no boundaries are crossed.

2) The boundaries are indistinct, and spike and cell migration as a cell population toward the outer boundary were observed.

Fluorescence images were obtained using confocal microscopy (TCS SPE; Leica Microsystems, Wetzlar, Ger-many). The control soft gels without Pt (II) ions were constructed by the commercial 0.1 wt% type I collagen solution by neutralization and heating at 37 °C using a collagen gel culturing kit (Cellmatrix®, Nitta Gelatin Inc., Osaka, Japan). The images of the categories were indicated in Figure S5.

**Statistical analysis**

All data were expressed as means ± SD unless otherwise specified. The values represent the mean ± SD from more than three independent experiments. Statistical comparisons between groups were analyzed by one-way ANOVA using GraphPad Prism 9 (GraphPad software, San Diego, CA).

A *p* value, * <0.05, ** <0.01, ***<0.001, and ****<0.0001 were considered to be statistically significant. N.S. means no significant difference.

**S2. Effect of TM species on gelation**

**Table S1.** Effect of metal species on gelation property of Col-TM gels fabricated by mixing 0.5 wt% CNF and 0.5 mM TM ions.

| Group | Metal species | | | Gelation | Gelation time | Elastic modulus (kPa) |
|---|---|---|---|---|---|---|
| 2 | Mg (II) | | $MgCl_2$ | × | — | — |
| | Ca (II) | | $CaCl_2$ | × | — | — |
| | Ba (II) | | $BaCl_2$ | × | — | — |
| 8 | Fe (III) | | $FeCl_3$ | × | — | — |
| 9 | Co (II) | | $CoCl_2$ | × | — | — |
| | Ir (IV) | | $IrCl_4$ | × | — | — |
| 10 | Ni (II) | | $NiCl_2$ | ○ | 30 min | 136 |
| | Pd (II) | | $PdCl_2$ | △ | 20 s | 46 |
| | Pt | (II) | $K_2PtCl_4$ | ○ | 3 min | 1034 |
| | | | $Pt(NH_3)_4Cl_2$ | ○ | 3 min | 1670 |
| | | (IV) | $K_2PtCl_6$ | ○ | 20 min | 300 |
| 11 | Cu (II) | | $CuCl_2$ | △ | 1 min | 36 |
| | Ag (I) | | $AgNO_3$ | × | — | — |
| | Au | (0) | | × | — | — |
| | | (III) | $HAuCl_4$ | ○ | 10 s | 560 |
| 12 | Zn (II) | | $ZnCl_2$ | △ | 20 min | 33 |

**S3. S-S curve of Col-Pt gels and CD spectra of Col-Au gels and denatured collagens**

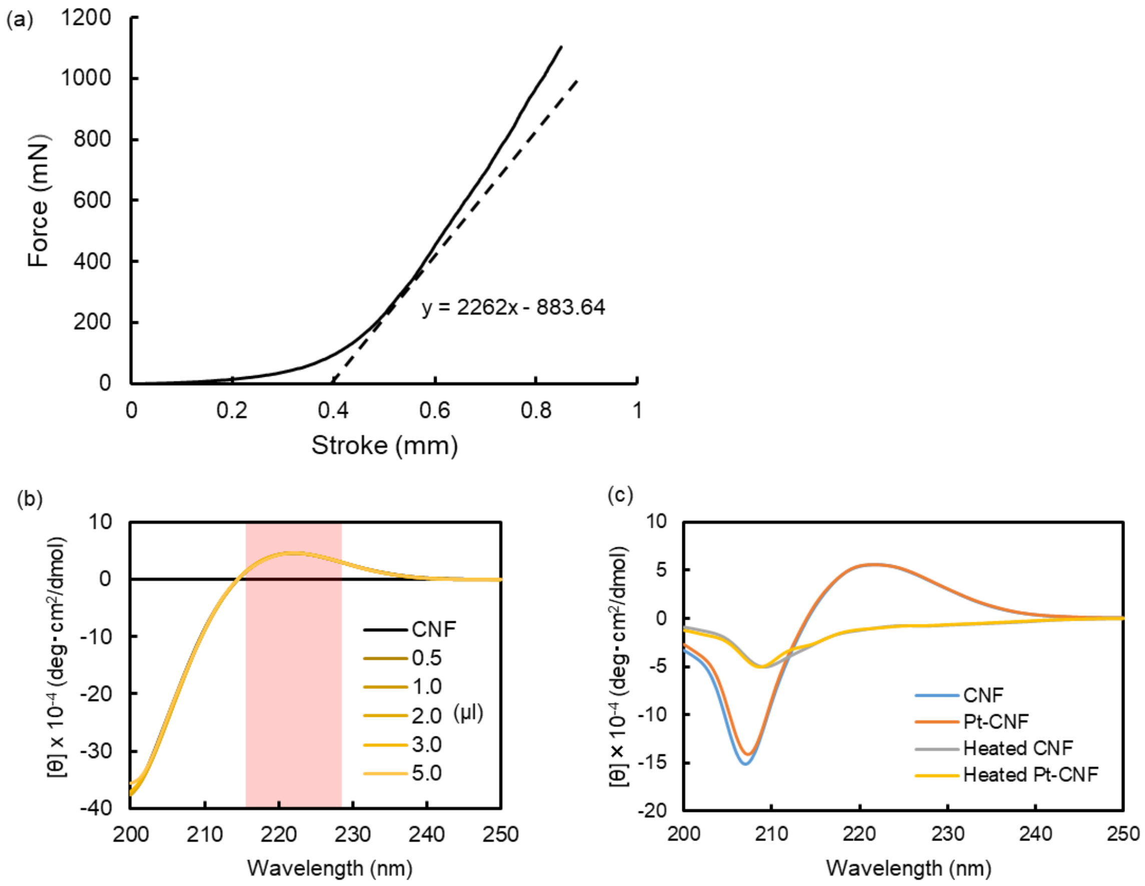


**Figure S1.** (a) Stress-strain curve of the obtained gels by mixing 1.0 wt% CNF and 0.1 mM $K_2Pt(II)Cl_4$ after the incubation at 37 $^oC$ for 1 day in 24 well insert. (b) CD spectra of CNF solution with different concentration of Au(III) at room temperature. (c) CNF and thermally-denatured CNF with or without $K_2Pt(II)Cl_4$ by heating at 80 $^oC$ for 30 min.

**S4. Effect of collagen species and type on gelation**

**Table S2.** Effect of collagen species and type on gelation property of Col-Pt gels.

| Species | Type | Triple helix | Gelation |
|---|---|---|---|
| Pig | Col I | ○ | ○ |
| | Col III | ○ | ○ |
| | Tropo Col I | ○ | ○ |
| Chicken | Col II | ○ | ○ |
| Bovine | Col I | ○ | ○ |
| Human | Col I | ○ | ○ |
| | Col IV | ○ | ○ |

## S5. Effect of polymer type on gelation

**Table S3.** Effect of polymer type on gelation property of Col-Pt gels.

| Polymer | $M_W$ | Helix | Gelation |
|---|---|---|---|
| Gelatin | 100,000 | × | × |
| $(POG)_{10}$ | 2,700 | ○ | × |
| Laminin | 865,000 | × | × |
| Fibronectin (0.1 wt%) | 250,000 | × | × |
| Heparin | 12,000 | × | × |
| Alginate | – | × | × |
| Polyacrylate | 250,000 | × | × |
| DNA | – | ○ | × |

**S6. Effect of gelatin concentration on gelation**

**Table S4.** Effect of gelatin concentration on gelation property of Gel-Pt gels.

| Gelatin concentration (wt%) | Pt(II) concentration (mM) | Gelation |
|---|---|---|
| 5.0 | 1.0 | × |
| | 5.0 | × |
| | 10 | × |
| | 50 | × |
| 10 | 1.0 | × |
| | 5.0 | △ |
| | 10 | ○ |
| 20 | 1.0 | × |
| | 5.0 | ○ |
| | 10 | ○ |

**S7. Viscoelasticity of CNF and gelatin solutions**

**Table S5.** Viscoelasticity of CNF and gelatin solutions.

| Substance | wt% | Viscoelasticity (mPa·S) |
|---|---|---|
| CNF | 0.2 | 22 |
| | 0.5 | 121 |
| | 1.0 | 429 |
| Gelatin | 5.0 | 1.6 |
| | 10 | 5.0 |
| | 20 | 22 |

**S8. Pt $L_3$-edge X-ray absorption spectroscopy of Col-TM gels**

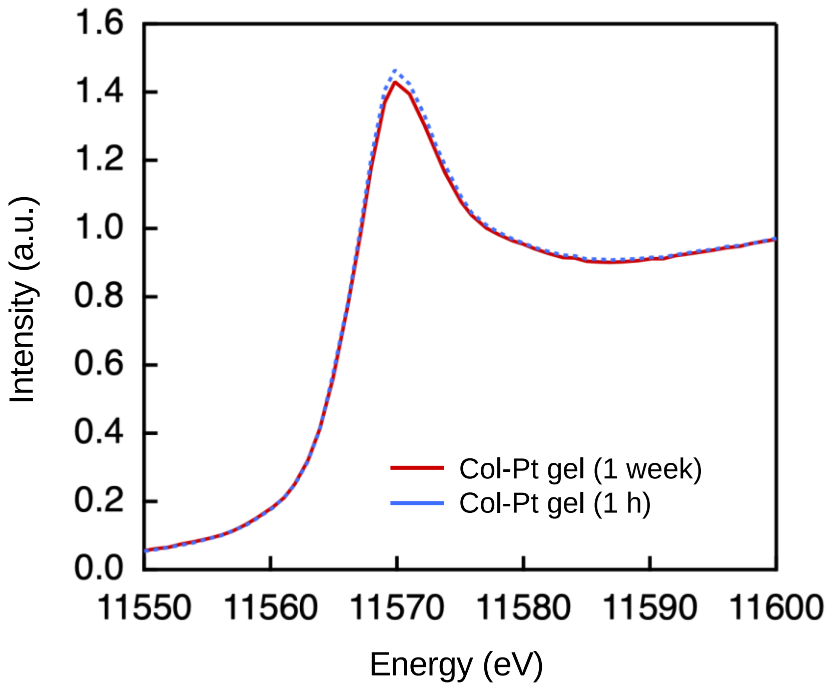


**Figure S2.** XANES spectra of Col-Pt gels after incubation for 1 h (blue dotted line) and 1 week (red line) at 4 °C

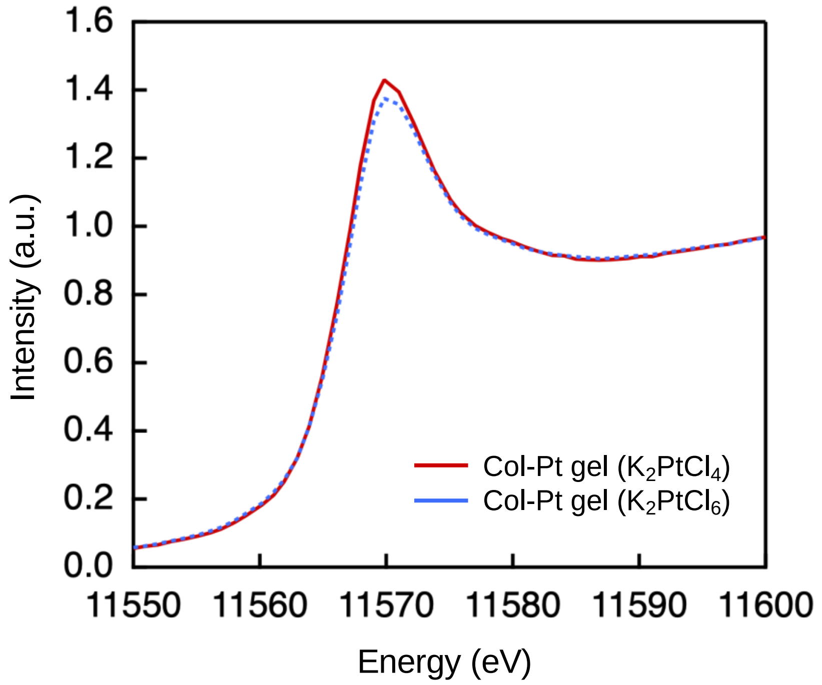


**Figure S3.** XANES spectra of Col-Pt gels prepared from $K_2PtCl_4$ (red line) and $K_2PtCl_6$ (blue dotted line).

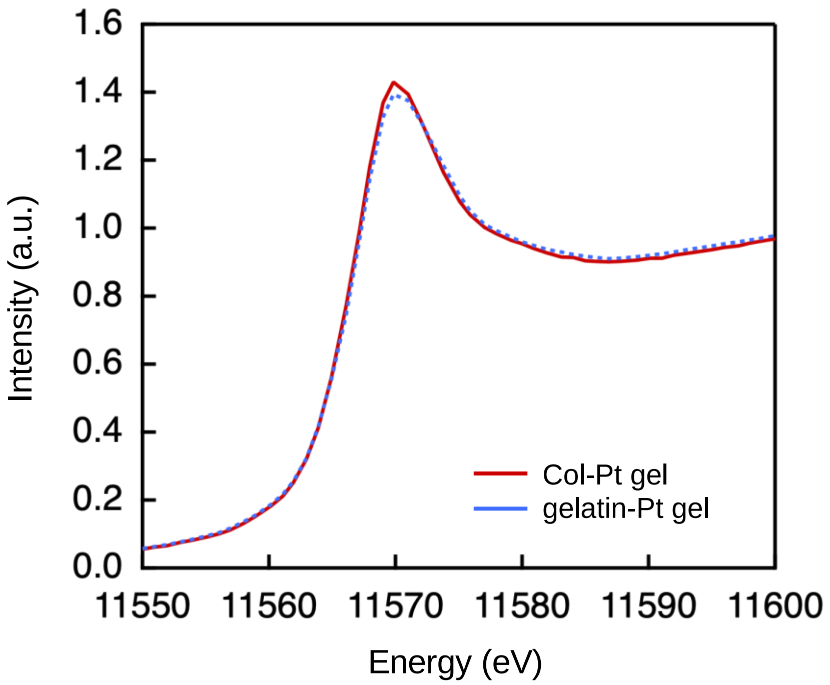


**Figure S4.** XANES spectra of Col-Pt gel (red line) and gelatin-Pt gel (blue dotted line).

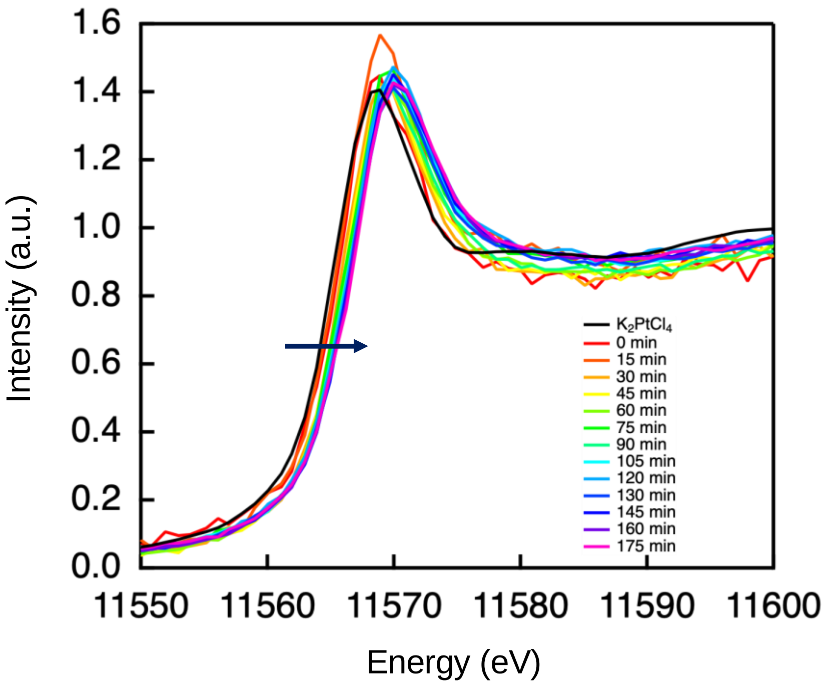


**Figure S5.** Time-course of XANES spectra of $(POG)_{10}$ + $K_2Pt(II)Cl_4$ in PBS. The times shown inset signify the start time of the measurements. XAS measurement takes approximately 15 min for 1 scan.

**S9. ICP data of Col-Pt gels**

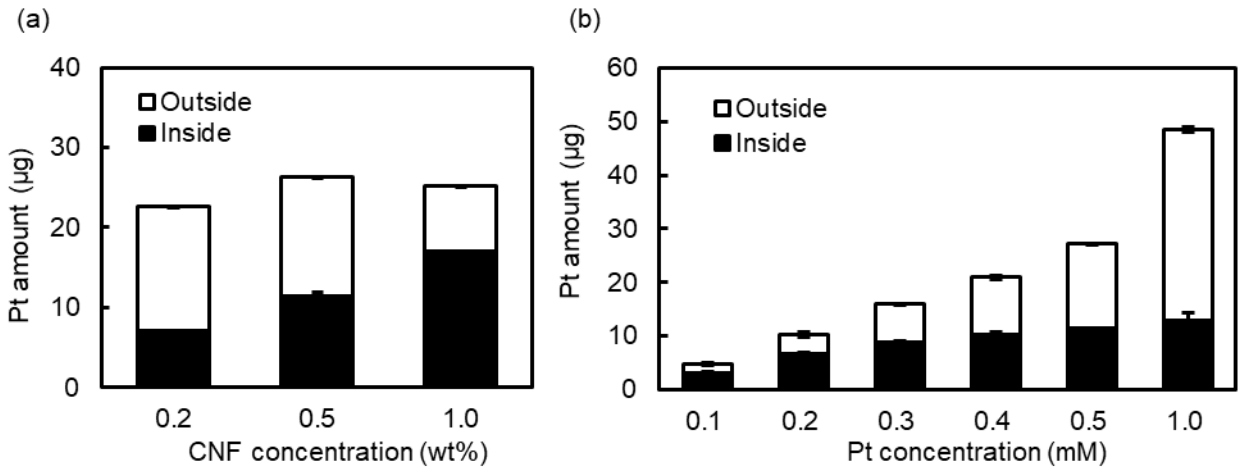


**Figure S6.** Estimation of the amount of Pt(II) ions inside and outside Col-TM gels (n=3). (a) Col-TM gels were prepared by (a) 0.5 mM $K_2Pt(II)Cl_4$ and 0.2–1.0 wt% CNF solutions and (b) 0.5 wt% CNF solution and 0.1–1.0 mM $K_2Pt(II)Cl_4$. The obtained gels were washed with 1 ml of PBS for 3 times and then measured the amount of Pt(II) ions inside the gels (inside) and whole washing PBS (outside).

**S10. Cell viability assay**

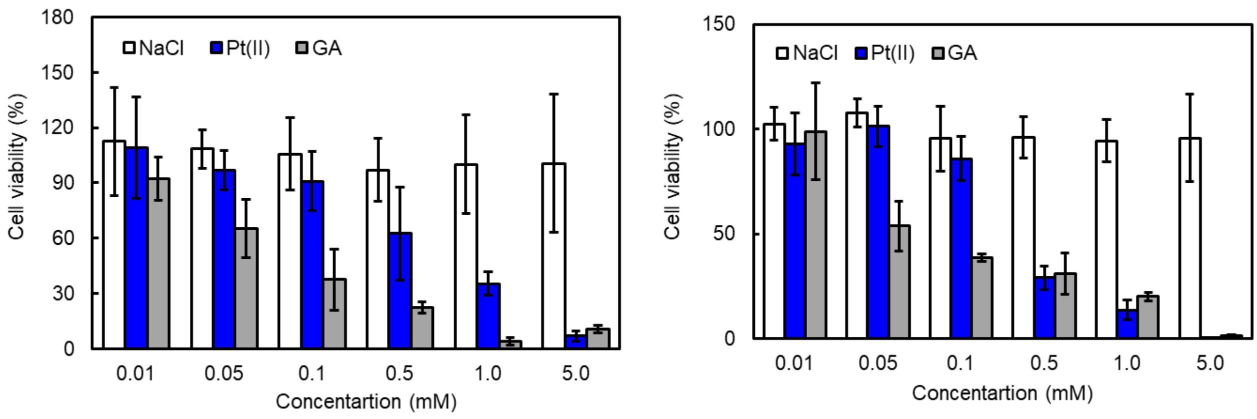


**Figure S7.** Cell viability of JC-011(left) and NHDFs (right) in DMEM containing 10% FBS and NaCl, glutaraldehyde (GA) or $K_2Pt(II)Cl_4$ for 3 days of culture without medium change (n=3).

**S11. 3D-culture of organoids in Col-Pt gels**

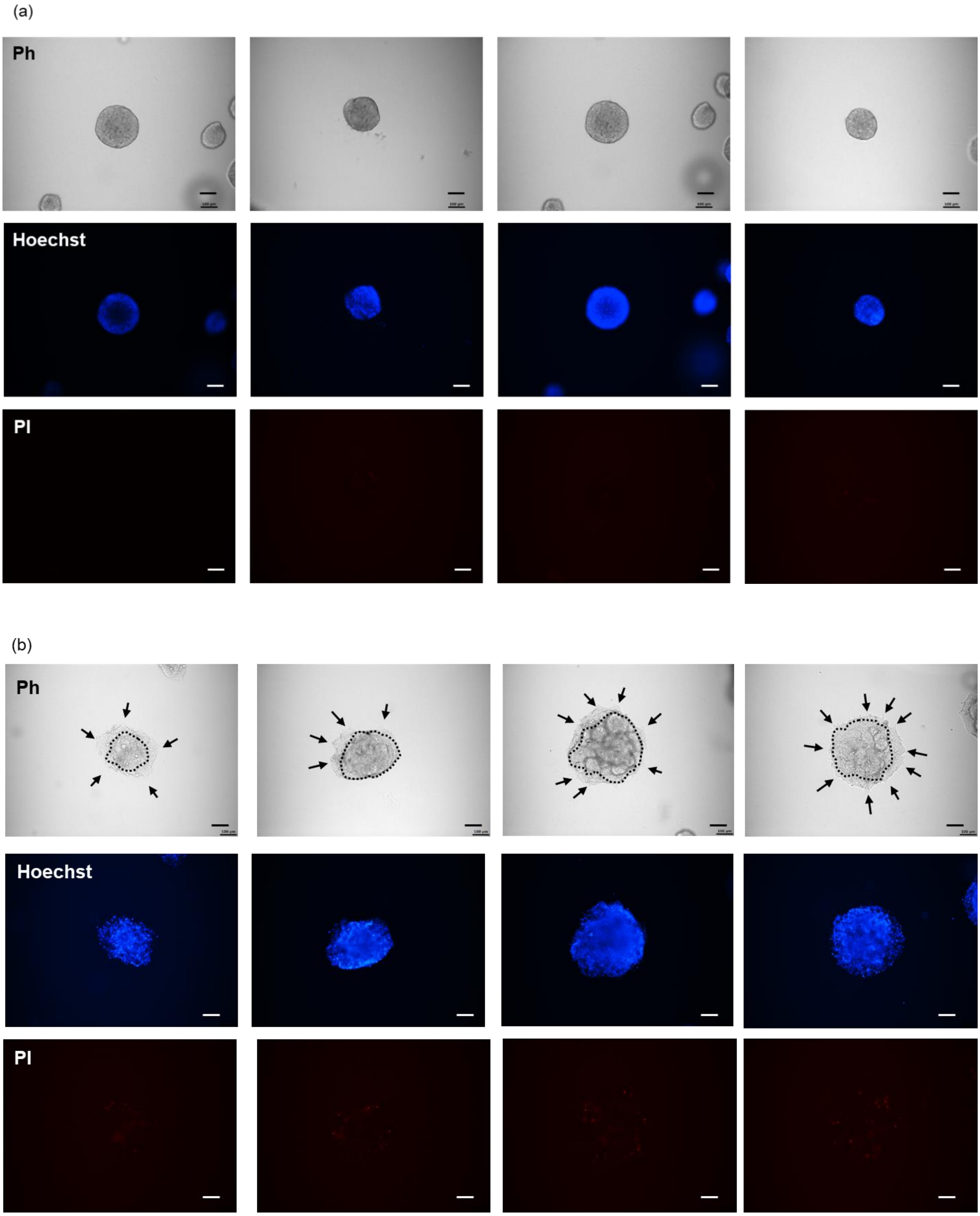


**Figure S8.** Phase contrast, Hoechst and PI images of CTOS cultured in (a) control soft collagen gels and (b) stiff Col-Pt gels constructed by 0.2 wt% CNF with 0.5 mM $K_2PtCl_4$ solutions. Black arrows and dashed lines indicate spike/cell migrations and boundary, respectively. Scale bars are 100 mm.

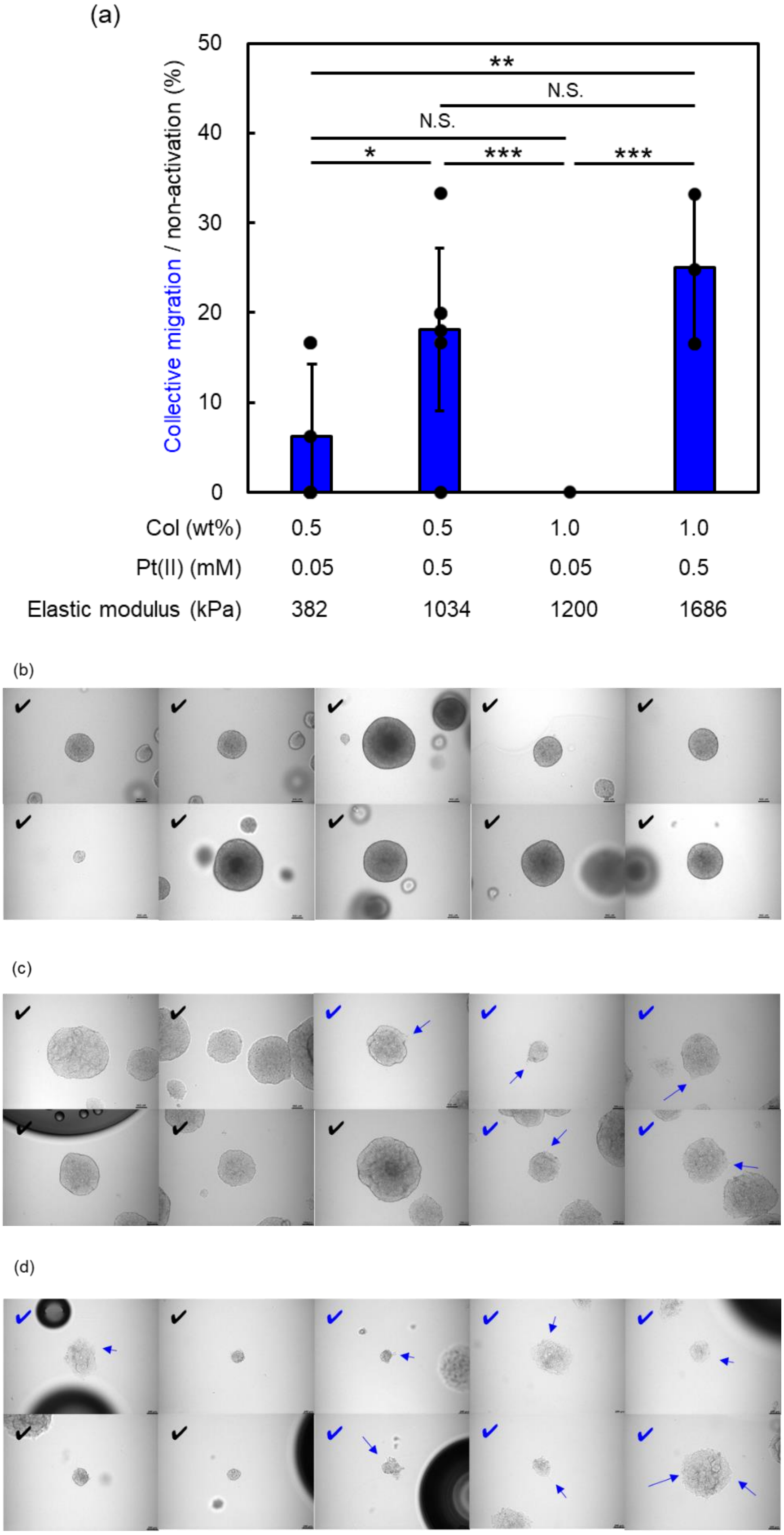
(a)
Collective migration / non-activation (%)
50
40
30
20
10
0
**
N.S.
N.S.
*
***
***
Col (wt%)
0.5
0.5
1.0
1.0
Pt(II) (mM)
0.05
0.5
0.05
0.5
Elastic modulus (kPa)
382
1034
1200
1686
(b)
(c)
(d)

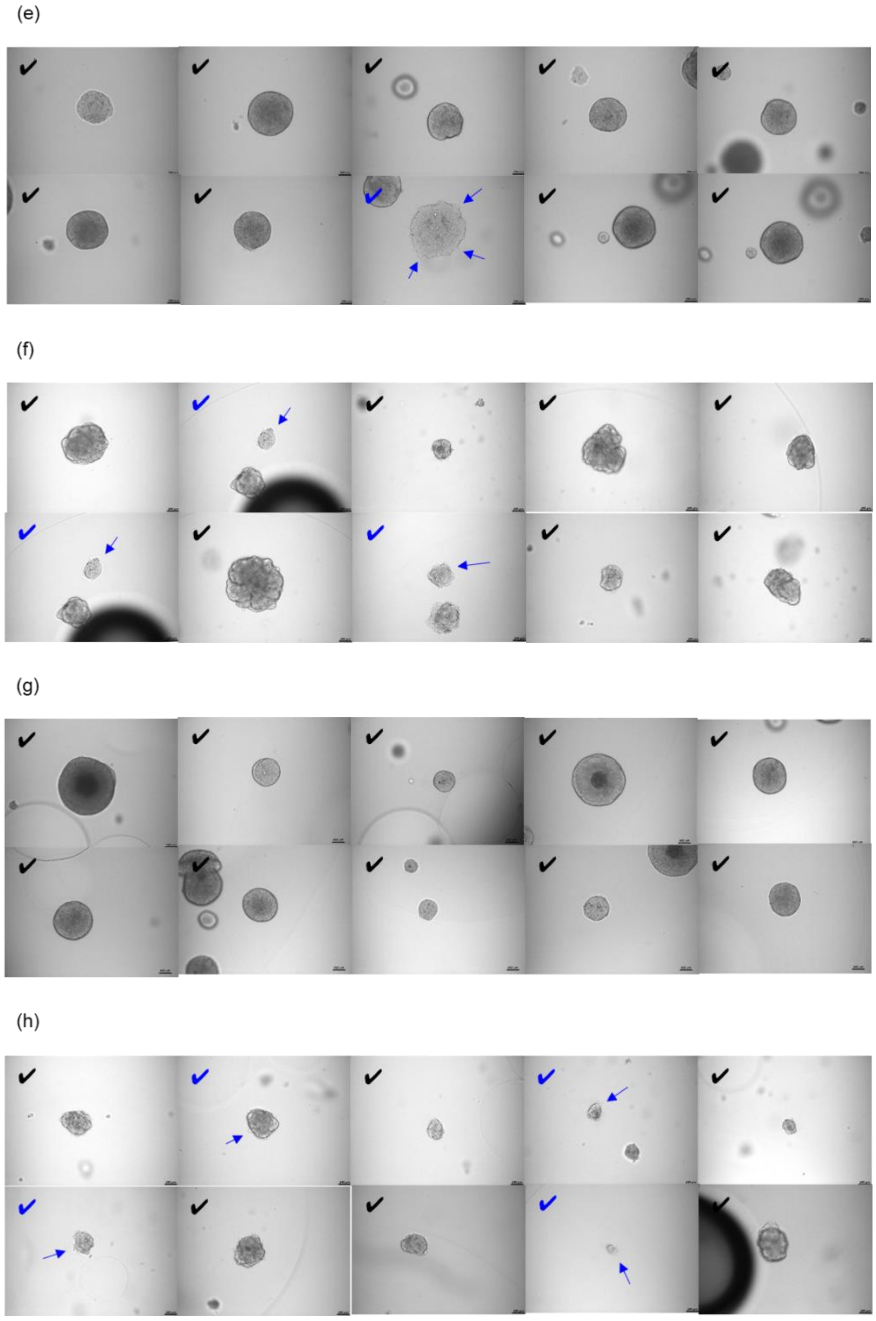


**Figure S9.** (a) Mean percentage of both collective migration and non-activation of CTOS after 3D culture inside the gels constructed by varied Col and Pt(II) concentrations (n=4~8). Phase contrast images of CTOS cultured in (b) control soft collagen gels and Col-Pt gels constructed by (c) 0.2 wt% CNF with 0.05 mM $K_2PtCl_4$ solutions, (d) 0.2 wt% CNF with 0.5 mM $K_2PtCl_4$ solutions, (e) 0.5 wt% CNF with 0.05 mM $K_2PtCl_4$ solutions, (f) 0.5 wt% CNF with 0.5 mM $K_2PtCl_4$ solutions, (g) 1.0 wt% CNF with 0.05 mM $K_2PtCl_4$ solutions, and (h) 1.0 wt% CNF with 0.5 mM $K_2PtCl_4$ solutions after 7 days of culture. Black and blue checks and arrows represent non-activation and activation of organoids, respectively. Scale bars are 100 mm. Statistical comparisons between

groups were analyzed by one-way ANOVA. A $p$ value, * <0.05, ** <0.01, and ***<0.001 were statistically significant. N.S. means no significant difference.

**S12. Gelation videos of Col-TM gels**

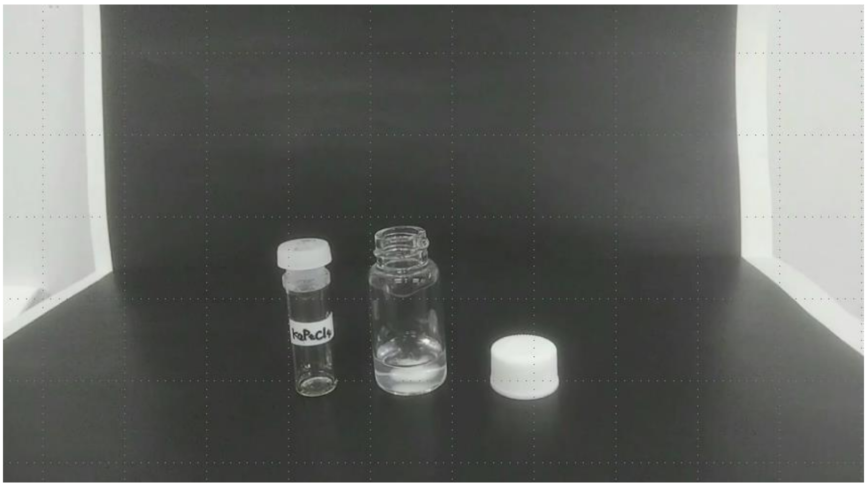

**Video S1.** Gelation video of 0.5 wt% CNF solution by mixing with 0.5 mM $K_2Pt(II)Cl_4$ at r.t.

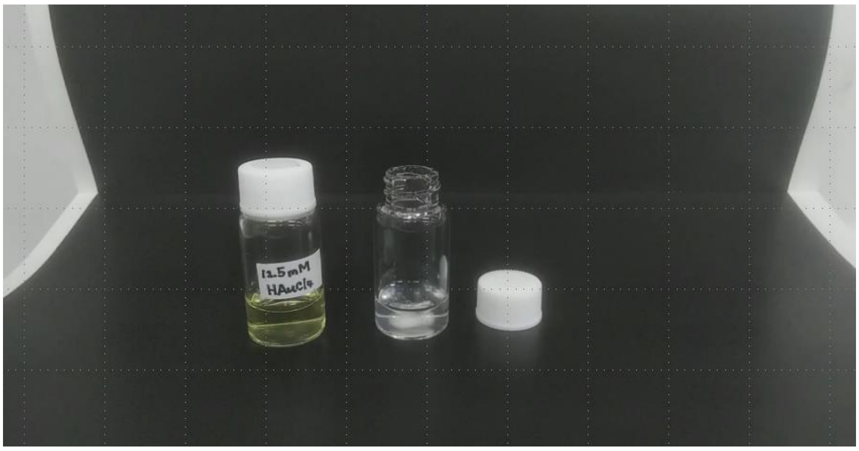


**Video S2.** Gelation video of 0.5 wt% CNF solution by mixing with 0.5 mM $HAuCl_4$ at r.t.

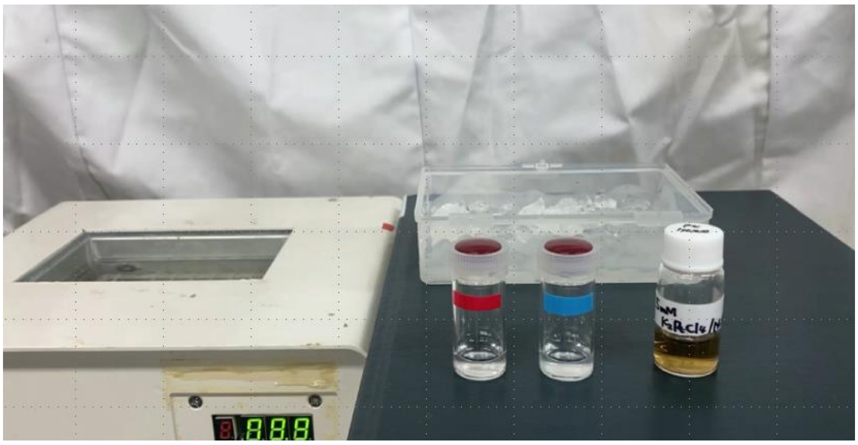

**Video S3.** Gelation comparison between 0.5 wt% CNF (blue vial) and 0.5 wt% gelatin (red vial) solutions by mixing with 0.5 mM $K_2Pt(II)Cl_4$ at r.t.

**S13. OCT observation**

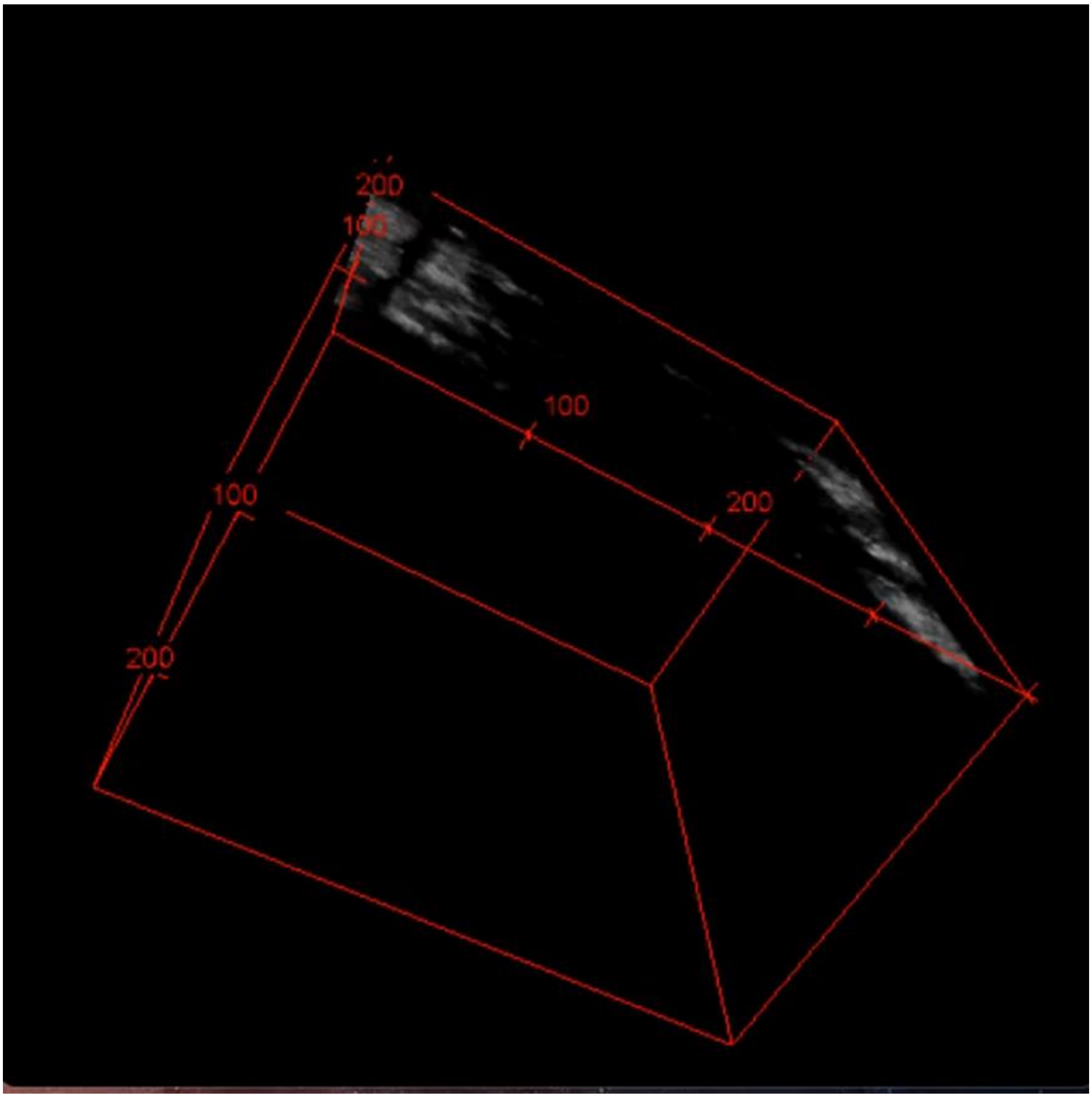


**Video S4.** 3D-rotating animation of OCT observation of Col-Pt gels constructed at 1.0 wt% CNF solutions by mixing with 0.5 mM $K_2Pt(II)Cl_4$ at r.t. for 1 day. Observation area is 4.0 mm and depth is 1.35 mm, respectively. Top surface of OCT image is bottom surface of the gels.

**S14. References**

[S1] J. J. Rehr, R. C. Albers, *Rev. Mod. Phys.* **72**, 621–654 (2000).
[S2] M. Newville, *J. Synchrotron Radiat.* **8**, 322–324 (2001).
[S3] B. Ravel, M. Newville, *J. Synchrotron Radiat.* **12**, 537–541 (2005).
